# Clinical utility of circulating tumor DNA as a response and follow-up marker in cancer therapy

**DOI:** 10.1007/s10555-020-09876-9

**Published:** 2020-05-04

**Authors:** Pieter A. Boonstra, Thijs T. Wind, Michel van Kruchten, Ed Schuuring, Geke A. P. Hospers, Anthonie J. van der Wekken, Derk-Jan de Groot, Carolien P. Schröder, Rudolf S. N. Fehrmann, Anna K. L. Reyners

**Affiliations:** 1 Department of Medical Oncology, University of Groningen, University Medical Centre Groningen, Hanzeplein 1, 9700 RB Groningen, The Netherlands; 2 Department of Pathology, University of Groningen, University Medical Centre Groningen, Hanzeplein 1, 9700 RB Groningen, The Netherlands; 3 Department of Pulmonary Medicine, University of Groningen, University Medical Centre Groningen, Hanzeplein 1, 9700 RB Groningen, The Netherlands

**Keywords:** ctDNA, Mutation detection, Therapy monitoring, Follow-up, Driver mutations

## Abstract

Response evaluation for cancer treatment consists primarily of clinical and radiological assessments. In addition, a limited number of serum biomarkers that assess treatment response are available for a small subset of malignancies. Through recent technological innovations, new methods for measuring tumor burden and treatment response are becoming available. By utilization of highly sensitive techniques, tumor-specific mutations in circulating DNA can be detected and circulating tumor DNA (ctDNA) can be quantified. These so-called liquid biopsies provide both molecular information about the genomic composition of the tumor and opportunities to evaluate tumor response during therapy. Quantification of tumor-specific mutations in plasma correlates well with tumor burden. Moreover, with liquid biopsies, it is also possible to detect mutations causing secondary resistance during treatment. This review focuses on the clinical utility of ctDNA as a response and follow-up marker in patients with non-small cell lung cancer, melanoma, colorectal cancer, and breast cancer. Relevant studies were retrieved from a literature search using PubMed database. An overview of the available literature is provided and the relevance of ctDNA as a response marker in anti-cancer therapy for clinical practice is discussed. We conclude that the use of plasma-derived ctDNA is a promising tool for treatment decision-making based on predictive testing, detection of resistance mechanisms, and monitoring tumor response. Necessary steps for translation to daily practice and future perspectives are discussed.

## Introduction

Response evaluation during anti-cancer therapy and follow-up of patients with solid malignancies is currently primarily based on radiological assessments according to response evaluation criteria in solid tumors (RECIST) [1]. Repeated radiologic assessments are however time consuming, costly, and increase the radiation burden for the patient. This is especially an issue in the context of the increasing number of long-term cancer survivors due to new anti-cancer therapies. Moreover, response evaluation based on radiologic assessment is problematic with certain novel therapies. For example, immunotherapy can cause pseudoprogression on radiologic assessments as a result of influx of cytotoxic T-lymphocytes [2]. Irradiation of high-grade glioma can cause pseudoprogression on MRI in approximately one-third of the patients [3]. And anti-VEGF therapy in colorectal cancer can result in morphological changes such as altered delineation of the tumor, which predicts pathologic response and overall survival better than does standard radiologic assessment according to RECIST [4]. Finally, response assessment can be difficult in certain settings regardless the therapy given. In a bone-dominant disease such as prostate cancer and hormone-positive breast cancer, response assessment is hampered as bone lesions are considered non-evaluable by RECIST [5].

Novel therapies may not only cause difficulties with regard to radiologic response assessment; these new treatments often also aim at specific mutations (i.e., receptor tyrosine kinases that are in a continuously activated state due to genetic aberrations). Therefore, for treatment decision-making up to date, information about the genomic composition of the tumor lesions is crucial. Frequently, archival tissue is used for genomic analysis of molecular aberrations. However, tumor characteristics can change during the course of disease, such as development of new mutations causing secondary resistance. Repeated biopsies may be obtained, but this is not always feasible, invasive, and not always representative of the whole tumor burden due to sampling error and tumor heterogeneity [6].

To circumvent the abovementioned limitations regarding radiologic response assessment, as well as the need for up-to-date information about molecular characteristics, there is a clinical need for tumor-specific, highly sensitive, non-invasive assays to determine the genomic composition of tumors and to assess response accurately in solid malignancies.

## Liquid biopsies

A potential method to obtain information about both the genomic composition of tumors and the tumor burden is through detection and quantification of tumor DNA in plasma. Tumor DNA can be identified by tumor-specific mutations that are derived from circulating tumor cells (CTCs), tumor-derived vesicles (exosomes), and nucleosome-bound tumor DNA that is shed into the circulation during necrosis or apoptosis of tumor cells [7–9]. Various methods to analyze and quantify circulating tumor DNA (ctDNA) are available [10–12]. First-generation sequencing methods are PCR-based techniques such as droplet digital PCR (ddPCR) and breads, emulsification, amplification and magnetics (BEAMing). Although PCR-based techniques are limited by evaluating only a low number of pre-specified mutations, the costs are relatively low, an absolute number of aberrant copies per mL can be provided, turnaround time is short, and sensitivity high. More recently, next-generation sequencing (NGS) has been developed, which can cover larger panels of selected genes/mutations, whole-exome or even whole-genome sequencing. Aside from its larger coverage when compared with ddPCR, NGS also has the advantage that mutations do not need to be pre-specified and therefore rare and novel mutations can be detected. However, NGS is more costly, turnaround time is longer, and sensitivity for mutations with low mutant allelic frequency can be lower than with ddPCR [13].

As a method to quantify tumor burden, liquid biopsy has the advantage over radiologic assessments that it may differentiate between pseudoprogression and true progression, may be used to evaluate response in settings in which radiologic assessment is difficult (such as bone-dominant disease), and can reduce radiation burden. As a method to obtain molecular information, liquid biopsy has the advantage over biopsy-driven genomic analysis that it is non-invasive, can provide information about presence of various subclones, and gives the opportunity to evaluate for secondary resistance mutations during the course of disease. At this moment, the evidence to support widespread use of ctDNA as a predictive or prognostic marker in patients with solid malignancies is limited [14]. In this review, we summarize data on the application of ctDNA analysis as a treatment response and follow-up marker in patients with solid malignancies. We focus on non-small cell lung carcinoma (NSCLC), melanoma, colorectal carcinoma (CRC), and breast cancer, given the specific driver mutations that are often present and the availability of targeted drugs.

## Search strategy and quality of included studies

A PubMed search was performed on January 1, 2019, using the following syntax: (Oncology[tiab] OR Cancer* [tiab] OR malignant[tiab] OR malignanc*[tiab] OR tumor[tiab] OR tumour [tiab]) AND (DNA[tiab] OR “ Deoxyribonucleic acid”[tiab] OR RNA[tiab] OR “Ribonucleic Acid”[tiab]) AND (Mutation*[tiab] OR Rearrange* [tiab]) AND ((“circulating”[tiab] OR ctDNA[tiab] OR cfDNA[tiab] OR “liquid biopsy” OR “blood based” OR “Circulating tumor cells”[tiab] OR “Circulating tumour cells”[tiab] OR CTC[tiab] OR (“platelets”[tiab] OR Thrombocytes[tiab])) AND (“humans”[MeSH Terms] AND English[lang]). The search was limited to full articles, written in English. In total, 1057 articles were identified. Articles were screened on title, abstract, and full text by PAB and TTW. Articles describing sequential ctDNA measurements in human patients with solid malignancies during systemic therapy were eligible. Studies regarding the use of CTCs, exosomes, or other circulating markers were excluded. Studies that investigated detection of mutations in body fluids other than plasma were not within the scope of this review.

Finally, 82 articles were eligible for this review (Table 1). Of these, 26 articles provided detailed descriptions of individual cases or case series. No randomized clinical trials were available. The remaining 56 articles consisted of studies that evaluated the association of plasma ctDNA levels with response rate (RR), progression-free survival (PFS), and/or overall survival (OS). Relevant articles that not matched our search criteria were occasionally added. All papers were classified for level of evidence following the rules as depicted by the Oxford Centre for Evidence-Based Medicine [15]. Six studies were classified as exploratory cohort studies with good reference standards resulting in a score of 2b (2 melanoma and 4 CRC studies). Fifty studies were non-consecutive studies without consistently applied reference standards (3b) and 26 studies consisted of case reports or small series without poor or non-independent reference standards (4, Table 1). Although the largest study included 200 patients, most studies have low patient numbers (range 1–200, median 14 patients).

**Table 1:** Overview of the papers retrieved by the search and included for this review. All papers were classified for level of evidence following the rules as depicted by the Oxford Centre for Evidence-Based Medicine [15]

Author	Tumor type	Paper score	Gene of interest	Technique	Therapy	*N*	Disease status	Mutation detection rate in plasma	Predictive for disease progression	Predictive for response	Progression ctDNA before radiological
Alegre [16]	NSCLC	3b	EGFR	ddPCR	EGFR TKI	8	Metastasized	65%	Yes	Yes	-
Arulananda [17]	NSCLC	4	EGFR	ddPCR	EGFR TKI	1	Metastasized	-	Yes	Yes	-
Demuth [18]	NSCLC	3b	EGFR	ddPCR	EGFR TKI	144	Metastasized	100%	-	-	-
Guibert [19]	NSCLC	3b	KRAS	ddPCR	Multiple	16	Metastasized	78%	Yes	Yes	-
Guibert [20]	NSCLC	4	KRAS	ddPCR	Anti-PD-1	2	Metastasized	-	-	Yes	-
He [21]	NSCLC	3b	EGFR	ddPCR	EGFR TKI	128	Metastasized	93%	Yes	Yes	-
Iijima [22]	NSCLC	3b	Various	NGS	Anti-PD-1	14	Metastasized	23%	-	Yes	-
Imamure [23]	NSCLC	3b	EGFR	NGS	EGFR TKI	38	Metastasized	73%	Yes	Yes	-
Imamure [24]	NSCLC	3b	EGFR	NGS	EGFR TKI	21	Metastasized	66.60%	-	Yes	-
Iwama [25]	NSCLC	3b	EGFR	ddPCR, NGS	EGFR TKI	32	Metastasized	81%	Yes	Yes	-
Jia [26]	NSCLC	3b	EGFR + KRAS	ddPCR	Not specified	150	Metastasized	89%	Yes	Yes	Unknown
Jiang [27]	NSCLC	3b	TP53	Seq	Chemotherapy	28	Metastasized	100%	Yes	Yes	-
Jovelet [28]	NSCLC	4	EGFR	ddPCR	EGFR TKI	7	Metastasized	62%	Yes	Yes	-
Knebel [29]	NSCLC	4	EGFR	ddPCR	EGFR TKI	1	Metastasized	-	Yes	Yes	Yes
Lee [30]	NSCLC	3b	EGFR	ddPCR	EGFR TKI	40	Metastasized	74%	Yes	Yes	Yes
Liang [31]	NSCLC	4	EML4 - ALK, TP53	Seq	ALKi	1	Metastasized	-	Yes	Yes	-
Minari [32]	NSCLC	4	EGFR	ddPCR	EGFR TKI	5	Metastasized	100	-	Yes	-
Mok [33]	NSCLC	3b	EGFR	PCR	EGFR TKI	98	Metastasized	75%	Yes	Yes	-
Nakamura [34]	NSCLC	4	EGFR	PCR	EGFR TKI	2	Metastasized	45%	Yes	Yes	-
Dowler Nygaard [35]	NSCLC	3b	KRAS	PCR	Chemotherapy	7	Metastasized	-	Yes	Yes	-
Oxnard [36]	NSCLC	4	EGFR, BRAF	PCR	EGFR TKI	4	Metastasized	50–81%	Yes	Yes	Yes
Pecuchet [37]	NSCLC	3b	EGFR, KRAS, BRAF	NGS, ddPCR	Multiple	85	Metastasized	71%	Yes	Yes	-
Piotrowska [38]	NSCLC	3b	EGFR	BEAMing	EGFR TKI	12	Metastasized	-	Yes	Yes	-
Punnoose [39]	NSCLC	3b	EGFR, KRAS, BRAF, PIK3CA	PCR	Pertuzumab, EGFR TKI	7	Recurrence	-	Yes	Yes	-
Riediger [40]	NSCLC	3b	EGFR + KRAS	ddPCR	EGFR TKI	16	Metastasized	93.70%	Yes	Yes	Yes
Seki [41]	NSCLC	3b	EGFR	ddPCR	EGFR TKI	15	Metastasized	71%	Yes	No	-
Sueoka-Aragane [42]	NSCLC	3b	EGFR	MBP-QP	EGFR TKI	58	Metastasized	40%	Yes	Yes	-
Thress [43]	NSCLC	3b	EGFR	NGS, ddPCR	EGFR TKI	19	Metastasized	40%	Yes	Yes	-
Uchida [44]	NSCLC	3b	EGFR	MPS	EGFR TKI	10	Metastasized	75%	Yes	Yes	-
Watanabe [45]	NSCLC	3b	EGFR	PCR	EGFR TKI	30	Metastasized	79%	Yes	Yes	-
Weber [46]	NSCLC	4	EGFR	PCR	EGFR TKI	1	Metastasized	-	-	Yes	-
Wei [47]	NSCLC	3b	EGFR	ddPCR	EGFR TKI	200	Metastasized	84%	Yes	Yes	-
Yu [48]	NSCLC	3b	EGFR	BEAMing	EGFR TKI	46	Metastasized	86%	Yes	Yes	-
Zheng [49]	NSCLC	3b	EGFR	ddPCR	EGFR TKI	55	Metastasized	81%	Yes	Yes	-
Zhou [50]	NSCLC	3b	EGFR	qPCR	EGFR TKI	80	Metastasized	-	No	Yes	-
Zhu [51]	NSCLC	3b	EGFR	ddPCR	EGFR TKI	48	Metastasized	81%	Yes	Yes	Yes
Ashida [52]	Mel	4	BRAF	castPCR	Multiple	6	Metastasized	50%	Yes	Yes	-
Casadevall [53]	Mel	4	BRAF	castPCR	BRAF-i	1	Metastasized	-	Yes	Yes	-
Chen [54]	Mel	3b	BRAF	RT-PCR, WES	BRAF-i	20	Metastasized	-	Yes	Yes	-
Gray [55]	Mel	3b	BRAF	ddPCR	MAPKi, BRAF-i, immunotherapy	25	Metastasized	65%	Yes	Yes	Yes
Quereux [56]	Mel	4	BRAF	dPCR	BRAF, MEK-i	1	Metastasized	100%	No	Yes	Yes
Sanmamed [57]	Mel	2b	BRAF	ddPCR	BRAF-i	16	Metastasized	84%	Yes	Yes	-
Schreuer [58]	Mel	3b	BRAF	qPCR, ddPCR	BRAF-i	36	Metastasized	70%	Yes	Yes	-
Seremet [59]	Mel	4	BRAF, NRAS	ddPCR	Multiple	7	Metastasized	100%	Yes	Yes	Yes
Shinozaki [60]	Mel	2b	BRAF	RT-PCR	Multiple	38	Various	37%	Yes	Yes	-
Arena [61]	CRC	3b	EGFR	ddPCR	Targeted therapy	2	Metastasized	18%	Yes	Yes	Yes
Bardelli [62]	CRC	4	KRAS, MET	PCR	EGFR TKI	1	Metastasized	-	Yes	No	Yes
Berger [63]	CRC	2b	KRAS	ddPCR	Chemotherapy	27	Metastasized	-	Yes	Yes	-
Carpinetta [64]	CRC	4	Various	NGS, ddPCR	Chemotherapy	4	Localized	-	Yes	Yes	Yes
Diehl [65]	CRC	3b	APC/KRAS/PIK3CA/ TP53	BEAMing	Chemotherapy	11	Various	-	Yes	Yes	-
Garlan [66]	CRC	2b	BRAF/KRAS/TP53	ddPCR	Chemotherapy	82	Metastasized	77%	No	Yes	No
Hong [67]	CRC	3b	BRAF	ddPCR	Multiple	12	Metastasized	-	Yes	Yes	-
Kakizawa [68]	CRC	3b	KRAS	ddPCR	Regorafenib	16	Metastasized	-	Yes	Yes	Yes
Khan [69]	CRC	3b	KRAS	ddPCR	Regorafenib	27	Metastasized	-	Yes	Yes	Yes
Oddo [70]	CRC	4	KRAS/BRAF/NRAS/EGFR/MAP2K1,2	NGS	BRAF-i, MEK-i	1	Metastasized	-	Yes	No	-
Russo [71]	CRC	4	MEK1/KRAS	NGS, ddPCR	Panitumumab, trametinib	1	Metastasized	-	Yes	Yes	No
Russo [72]	CRC	4	NTRK1,	NGS, ddPCR	Entrectinib	1	Metastasized	-	Yes	-	-
Siravegna [73]	CRC	4	CAD-ALK	PNA-PCR	ALK inhibitor	1	Metastasized	-	Yes	No	Yes
Spindler [74]	CRC	3b	KRAS, BRAF	qPCR	Chemotherapy	35	Metastasized	85%	Yes	Yes	Yes
Sun [75]	CRC	3b	KRAS, BRAF, NRAS	ddPCR	EGFR TKI	140	Metastasized	97%	Yes	Yes	-
Thierry [76]	CRC	3b	KRAS/NRAS/BRAF	qPCR	Folfox, dasatinib, cetuximab	42	Metastasized	88%	Yes	No	No
Tie [77]	CRC	3b	KRAS/APC/BRAF/TP53/NRAS/PIK3CA/SMAD	MPS	Chemotherapy	48	Metastasized	92%	Yes	Yes	Yes
Toledo [78]	CRC	3b	BRAF/PIK3CA	BEAMing	FOLFIRI-cetuximab	23	Metastasized	-	Yes	Yes	Yes
Vidal [79]	CRC	2b	KRAS	BEAMing	Chemotherapy, anti-EGFR	55	Metastasized	97%	Yes	Yes	Yes
Vietsch [80]	CRC	3b	Various	NGS	Chemotherapy	10	Various	28–47%	-	-	-
Wong [81]	CRC	3b	KRAS/PIK3CA/BRAF	BEAMing	Regorafenib	14	Metastasized	40%	Yes	Yes	-
Yamada [82]	CRC	3b	KRAS	ddPCR	EGFR TKI	24	Metastasized	90%	Yes	Yes	Yes
Yamauchi [83]	CRC	2b	Various	PCR	Anti-VEGF	21	Metastasized	100%	Yes	No	-
Zeng [84]	CRC	4	PIK3CA	PNA-PCR	FOLFOX	6	Metastasized	100%	No	No	No
Chen et al. [85]	BC	3b	TP53	RT-PCR	Chemotherapy	6	Localized	-	Yes	Yes	-
Garcia-Saenz [86]	BC	3b	PIK3CA	ddPCR	Not specified	8	Stage IIB - IV	55%	Yes	Yes	-
Guttery [87]	BC	3b	ESR1, TP53	NGS, ddPCR	Endocrine therapy	11	Metastasized	36%	Yes	-	-
Jansen [88]	BC	4	Various	NGS	Tamoxifen	1	Metastasized	-	Yes	-	Yes
Ma [89]	BC	3b	Various	NGS	TKI	18	Metastasized	50%	Yes	-	-
Murtaza [90]	BC	4	Various	Seq	Multiple	1	Metastasized	-	Yes	**-**	**-**
Nakagomi [91]	BC	4	TP53	NGS	Chemotherapy	1	Metastasized	-	Yes	Yes	-
Page [92]	BC	3b	ESR1, TP53, PIK3CA	NGS, ddPCR	Multiple	9	Metastasized	50%	Yes	Yes	-
Parsons [93]	BC	4	Various	NGS	Targeted treatment	26	Metastasized	92%	Yes	Yes	-
Riva [94]	BC	3b	TP53	ddPCR	Chemotherapy	36	Localized	75%	Yes	Yes	-
Sefrioui [95]	BC	4	ESR1	ddPCR	Endocrine therapy	2	Metastasized	67%	Yes	Yes	Yes
Takeshita [96]	BC	4	ESR1	ddPCR	Multiple	13	Metastasized	46.2%	-	-	-
Wang [97]	BC	3b	ESR1	ddPCR	Endocrine, chemotherapy	4	Metastasized	24%	Yes	Yes	-

*BC*, breast cancer; *Mel*, melanoma; *CRC*, colorectal cancer; *NSCLC*, non-small cell lung cancer; *PCR*, polymerase chain reaction; *RT-PCR*, real-time PCR; *ddPCR*, droplet digital PCR; *BEAMing*, beads, emulsions, amplification, magnetics; *qPCR*, quantitative PCR; *MBP-QP*, mutation-based PCR - quenching probe; *castPCR*, competitive allele-specific Taqman PCR; *PNA-PCR*, peptide nucleic acid PCR; *Seq*, sequencing; *NGS*, next-generation sequencing; *WES*, whole-exome sequencing; *MPS*, massive parallel sequencing, *N*, number of patients for monitoring; - not reported

### Non-small cell lung cancer

The mutations of interest in most studies regarding NSCLC are effecting the epidermal growth factor receptor (EGFR). Of all EGFR mutations described in this review, 99% is found in NSCLC. Other genes in which mutations were observed frequently in NSCLC were TP53 and KRAS. Detection rate of primary EGFR mutations in pre-treatment plasma ranged between 23 and 100%, highest detection was reached with PCR-based methods compared with techniques based on (next-generation) sequencing (median 79% *vs* 66.6%, respectively).

Thirty-three of the included 35 studies showed a positive relation between treatment response and a decline in mutant fraction after initiation of treatment. Disease progression could be detected with ctDNA in 28 studies; 6 studies did not have follow-up long enough for detection of progressive disease and in one study, the decline in mutant ctDNA fragments did not correspond with clinical disease status (Table 1) [50].

Prolonged PFS was observed for patients with undetectable levels of ctDNA during treatment *versus* patients with persistent detectable levels of ctDNA compared with baseline levels [30, 33, 37]. A decrease or even disappearance of mutant EGFR after start of treatment is a prognostic factor and indicator of response and is associated with longer OS [21, 24, 47, 48, 51]. An increase of the EGFR activating mutation is suggestive for therapy resistance and subsequent disease progression [16, 25, 32]. Smaller studies and case reports presented similar results [27, 35, 44]. The use of ctDNA as an early response marker is implicated by a longer OS in patients with undetectable levels of ctDNA after 6 to 12 weeks of anti-EGFR therapy compared with patients with detectable levels of ctDNA after the same treatment period [30, 33, 37, 43, 46].

In patients with acquired EGFR tyrosine kinase inhibitor (TKI)–resistant NSCLC, a rise of primary EGFR-mutated DNA occurred simultaneously with the detection of new mutations in the plasma in the majority of the tested patients during treatment [28, 38, 41, 51]. Detection of the therapy-resistant T790M mutation during treatment is suggestive for disease progression and a worse OS [26, 34, 36, 42, 45, 49]. Secondary treatment-resistant mutations can also be used for treatment monitoring but occur at lower frequencies than the primary mutation and are therefore less suitable for detection of disease progression [40]. Furthermore, these secondary mutations could almost only be detected in patients with a primary EGFR mutation [18]. New uncommon mutations that developed during treatment indicate clonal heterogeneity of the tumor and could be detected using sequencing; this is shown by the detection of a novel C797S or L747P mutation and EML4-ALK gene translocation additional to the primary EGFR exon 19– or T790M-resistant mutation during treatment [17, 31, 41, 43].

Five studies reported an earlier detection of progressive disease by ctDNA assessment as detected with conventional radiological imaging [23, 29, 30, 40, 51].

KRAS mutations can also be used as circulating marker in NSCLC patients treated with chemotherapy; patients with a detectable KRAS mutation had worse overall survival compared with patients with wild-type DNA (median 3.6 *vs* 8.4 months, respectively) [35]. A detectable KRAS mutation also indicated resistance to treatment with EGFR-targeted therapy in those patients (i.e., erlotinib or pertuzumab) [19, 39]. Of interest is the recent development of a specific KRAS inhibitor that can target *KRAS*^*G12C*^ mutation [98].

When treatment with novel agents as nivolumab (anti-PD-1) was initiated, a decrease in detectable specific mutations in plasma within 8 weeks after start of therapy was observed in responders (*n* = 11), while in non-responders (*n* = 5) a stable or increasing level of plasma ctDNA was detected [20, 22].

### Cutaneous melanoma

Mutations in cutaneous melanoma were primarily observed in v-Raf murine sarcoma viral oncogene homolog B (BRAF). Detection rate of primary mutations in plasma ranged between 37 and 100% (median 70%); only one study used a sequencing approach to detect mutations (Table 1).

Two studies described a total of 31 patients with BRAF-mutated melanoma treated with BRAF-inhibitors (BRAF-i) alone or in combination with mitogen-activated protein kinase inhibitors (MEK-i) [54, 58]. A disease control rate (DCR) of 75% was found in patients in whom mutation copy levels in ctDNA decreased compared with a DCR of 18% in patients with a stable or increasing level of ctDNA after 8 days of therapy [54]. Patients with undetectable ctDNA levels after a median of 13 days (range 6–40) of BRAF-i therapy had longer PFS compared with patients with persistent detectable ctDNA levels during therapy (*n* = 36 in total) [58]. Other studies in patients with metastatic melanoma treated with BRAF-i alone or in combination with MEK-i described similar observations [52, 53, 55–57].

Seremet et al. described 7 patients treated with an immune checkpoint inhibitor (ICI) in which the course of treatment was reflected by changes in ctDNA in patients with BRAF- or NRAS-mutated disease [59]. After initiation of treatment, the mutant BRAF/NRAS copy level decreased and remained low or undetectable during complete response and increased in the case of progressive disease. However, another study in 15 patients reported no difference in ctDNA plasma levels after 4 to 8 weeks of ICI therapy in 13 patients compared with pre-treatment levels although only four patients responded to treatment (of which two had a 10-fold reduction in ctDNA levels) [55].

Finally, in 20 patients treated with a combination of dacarbazine, cisplatin, vinblastine, and tamoxifen, BRAF mutant copies were detected in plasma at baseline and could only be detected in the plasma of 1 out of 10 responders and in 7 out of 10 non-responders [60]. There were no studies reporting on the detection of new acquired mutations during treatment.

The introduction of BRAF-targeted and ICI therapy for patients with metastatic melanoma has led to an increase in OS [99]. In patients with irresectable cutaneous melanoma treated with ICI therapy, a major challenge is the differentiation between “true” progression and pseudo progression (occurring in ~ 10% of patients) on radiological response evaluation. Although other markers, such as serum s100B, LDH, and the immune-related response criteria, for radiological response assessment provide some guidance, no marker is currently available. In a recent study, plasma samples obtained from 29 patients with cutaneous melanoma who showed progression of disease after 12 weeks of ICI therapy, all patients with pseudo progression (*n* = 9) had undetectable or > 10-fold decrease in ctDNA levels compared with pre-treatment levels [100]. Conversely, of the patients with “true” progression (*n* = 20), 90% had stable or increasing ctDNA levels compared with pre-treatment levels after 12 weeks of ICI therapy.

Recent studies have shown an improvement of recurrence-free survival in patients with stage III melanoma treated with surgery followed by adjuvant treatment with an ICI [101]. However, ICI therapy bears potential long-lasting risks such as immune-related adverse events, a proportion of patients will be treated in vain and therapy costs are high [102, 103]. Therefore, selection of patients at risk for recurrence is of great importance.

### Colorectal cancer

In colorectal cancer, most studies concern mutations in KRAS. The detection rate of primary mutations in plasma was reported in 10 studies which all used PCR-based techniques. The presence of KRAS mutations ranged between 18 and 100% (median 89%).

A higher response rate to chemotherapy and a longer PFS is described in patients in whom a decrease in ctDNA levels during therapy was observed compared with patients with stable or increasing ctDNA levels during treatment [69, 77]. Although the studies showed a trend towards longer survival and better response rates in patients with decreasing or undetectable ctDNA levels upon treatment, no statistically significant association between ctDNA level, OS, PFS, or radiological response has been described [61, 63, 67, 70–72, 81]. A decrease in total circulating cell-free DNA (cfDNA) copies/ml and mutant KRAS/BRAF/TP53 levels after two cycles of therapy compared with baseline and a subsequent increase at the time of progression in patients with CRC were related to treatment response as well as resistance. The decrease after initiation of treatment was larger in responding than in non-responding patients [66, 74].

Resistance to EGFR-targeted treatment can be caused due to amplification of the MET proto-oncogene and mutations in PIK3CA. This MET amplification is reported to be detected in ctDNA before relapse is clinically evident [62, 84]. Mutations that are newly detected during treatment might reveal the rise of minor tumor clones that show resistance to the administered therapy [83].

The emergence of KRAS mutations in KRAS wild-type patients during anti-EGFR therapy is suggestive for disease progression and was in some studies detectable in the blood prior to radiographic detection of progressive disease [68, 75, 78, 79].

Three studies described differences in ctDNA levels in a total of 29 patients with CRC before and after surgery [64, 65, 82]. In all patients with a complete resection (*n* = 26), a decline in ctDNA levels in plasma was observed. Three patients had tumor recurrence, which occurred simultaneously with recurrence of a KRAS mutation in ctDNA. In cases without complete resection (*n* = 3), ctDNA levels decreased only slightly or even increased. Additionally, it was observed that in patients with disease recurrence, an increase of plasma ctDNA levels occurred before or at the same moment the CEA levels increased and 2–3 months before radiologic evaluation showed signs of recurrence [76, 82, 104]. The ctDNA status at postoperative day 30 could be indicative for disease recurrence. Of 94 patients, 10 patients had positive ctDNA samples at day 30 and had a significantly higher recurrence rate (70%) compared with patients without detectable ctDNA (11.9%) at day 30 [105].

Early detection of recurrence will increase the proportion of patients who are potentially eligible for curative therapy. A survival benefit from such an approach has been shown in several meta-analyses [106].

Another study that used sequencing for analysis of ctDNA described an increase of 34% in the amount of different detectable mutations at the time of progression [80]. These mutations were not detectable at the time of primary disease, indicating clonal evolution of the disease. Furthermore, NGS can be used to detect new emerging mutations in the ALK kinase during treatment with the ALK inhibitor entrectinib [73]. The emerged mutations are associated with treatment resistance and warrant treatment with second-generation ALK inhibitors.

### Breast cancer

TP53-mutations (*n* = 81), ESR1 (*n* = 82), PIK3CA-mutations (*n* = 53), and AKT-mutations (*n* = 31) have most frequently been assessed to evaluate response to therapy using ctDNA in patients with breast cancer. As a large variety of mutations in breast cancer is present, NGS seems more feasible to detect mutations compared with ddPCR. Six of the 13 included studies used sequencing for the detection of mutations. The mutation detection rate ranged from 24 to 92% with a median of 50%.

Sequencing of PIK3CA and TP53 performed on ctDNA of 30 patients showed that changes in tumor burden correlated better with the height of plasma ctDNA levels compared with CA 15-3 [107]. Detection of TP53 seems feasible to monitor treatment response as a decrease of TP53 after initiation of treatment corresponded with response and an increase was a sign of relapse [91]. Patients with undetectable levels of ctDNA after one cycle of neoadjuvant chemotherapy had longer PFS and OS compared with patients in whom ctDNA remained detectable [85, 94]. In 28 patients with estrogen receptor positive (ER+) and BCL-2 (estrogen responsive gene responsible for survival which is overexpressed in 80% of primary ER+ breast cancer), positive metastatic breast cancer (MBC) treated with tamoxifen and venetoclax (BCL-2 inhibitor) treatment responses were shown to correlate with serial changes in ctDNA in plasma. A significant reduction of both ESR1 and PIK3CA mutations was observed within 28 days of treatment in all patients and it appeared that radiological progression was preceded by a rise in ctDNA [108]. Changing allelic fractions of ctDNA for any given mutation reflected response to therapy and disease progression in 7 patients [93]. Similar results were described in smaller studies [86, 90, 95–97].

Murtaza et al. described a patient with metastatic breast cancer (MBC) in which tumor site-specific mutations were identified implying heterogeneity of the tumor [90]. Sequencing of ctDNA showed that local progression of one tumor site coincided with an increase of the circulating abundance of mutations attributed to the lesion at that specific tumor site. This shows that ctDNA reflects dynamic alterations in size and activity of metastases at various tumor sites. This is supported by the findings of Page et al. which described rising cfDNA concentrations at the moment when PIK3CA/TP53/ESR1 mutations did not increase or resolved in the plasma [92]. The rise is probably caused by another clone that is shedding DNA into the blood that is not detected with the used ctDNA analysis method.

New mutations have been detected at the moment of progression which implicate acquired resistance to the treatment [88, 109]. It was shown that patients with endocrine therapy–resistant disease and detectable ESR1 mutations in ctDNA had longer PFS when treated with fulvestrant (*n* = 45) compared with patients treated with exemestane (*n* = 18). Conversely, in patients with wild-type ESR1, no difference in PFS was observed between both treatment arms. This suggests that ctDNA may direct choice of treatment in patients with resistant disease. In line with these observations, a meta-analysis of a combined total of 1530 patients with ER+ MBC showed shorter PFS for patients with a detectable ESR1 mutation in plasma ctDNA. Plasma ESR1 mutations were associated with shorter PFS after aromatase inhibitor–based therapy, but were not predictive of survival in patients treated with fulvestrant containing therapy [110]. Only three studies report data in comparison with the time of radiological assessment. In two of these studies, the ctDNA preceded detection of recurrence with CT and in one study, ctDNA analysis was as sensitive as the CT scan [88, 89, 95].

Several studies report the detection of novel mutations in PIK3CA and ESR1 during therapy in patients with MBC resistant to palbociclib and fulvestrant. These findings could also guide future treatment strategies to overcome resistance [87, 111, 112].

## Future perspectives

### Liquid biopsies to guide targeted therapy

The studies discussed in this review show that various targets that directly affect treatment decision-making, such as EGFR mutation in NSCL, BRAF mutation in melanoma, and KRAS mutation in CRC, can be detected by liquid biopsies. However, currently, only one liquid biopsy assay to guide treatment decision-making is FDA approved; the Cobas EGFR v2, which can be used as a companion diagnostic for EGFR mutations associated with progression of EGFR mutation–positive NSCLC [113]. Thus, translation towards clinical implementation of ctDNA testing and the availability of appropriate guidelines are urgently needed [114]. For EGFR mutation testing in NSCLC using plasma samples, External Quality Assessments (EQA) showed a need for quality improvements in clinical settings based on a high level of diagnostic errors [113, 115]. Despite the promising results in the last few years (this review), disadvantages of current ctDNA testing include limited sensitivity, restricted clinical utility, and loss of a direct link between a mutation and a given lesion [116]. Therefore, ctDNA testing in clinical practice needs to be further investigated and international consensus has to be reached on standardized operating procedures [14].

With regard to sensitivity of liquid biopsies, a broad range sensitivity for mutation detection is seen in the published studies. This could partly be related to the method of analysis since not all used methods have the same sensitivity or specificity. Moreover, the mutations in the reported studies are frequently solely detected in plasma and not necessarily compared with mutations detected in the tumor tissue. Therefore, negative ctDNA results could in fact be true-negative due to absence of the given mutation. Since negative results can be either a result of detection limit as well as true-negative results, it is questionable whether refrainment from treatment can be based purely on the absence of a mutation in ctDNA, and tissue-based analysis will likely remain the golden standard. In contrast, positive ctDNA results have shown high specificity in the different studies and may well be used to guide therapy.

Ideally, either prospective evaluation or retrospective testing of ctDNA analysis and its relation with treatment outcome from randomized studies is needed to show that the predictive value of liquid biopsies is comparable with that of the current gold standard of tissue-based molecular analysis. For the FDA-approved Cobas EGFR v2, for example, the observed benefit from erlotinib in the ENSURE trial was comparable for the patients that had a positive liquid biopsy when compared with tissue-positive patients [117, 118]. In addition, in the phase III EURTAC trial positive, negative and overall agreement between liquid biopsy results and tissue-based analysis for EGFR mutation was very high (94.2%, 97.5%, and 96.3%, respectively), and it had similar predictive value for benefit from erlotinib over chemotherapy [119]. Finally, also in the phase II AURA2 trial, it was shown that T790M positive patients by liquid biopsy had a high objective response rate to osimertinib [120].

Comparable trials showing predictive value of liquid biopsies in other tumor types and for other treatments are needed before liquid biopsies can be considered a replacement for repeated tumor biopsies. Currently, various liquid biopsy tests have been granted FDA breakthrough device designation, among which the FoundationOne Liquid, which captures 70 oncogenes in different tumor types, the Guardant360, which is a 73-gene panel to guide treatment decision in NSCLC, and Resolution HRD to determine aberrations in genes associated with homologous recombination deficiency.

### Additional value of liquid biopsies for response evaluation

Currently, no liquid biopsy test is approved for response evaluation during treatment, but the studies discussed in this review indicate that this is a promising field. Detection of progressive disease with ctDNA before radiological progression is reported in twenty-one studies in this review. Since progression by ctDNA is detected simultaneously with radiological progression in the majority of the other studies, it could possibly be used as a substitute for the latter. However, to reliably use ctDNA in daily practice instead of radiological imaging, a more consistent sensitivity has to be reached concerning the detection of predictive and resistant mutations in plasma. Especially cases where no mutations are detected in the plasma are unreliable and should be tested with more sensitive assays. Additionally, more studies are needed that correlate plasma mutations with radiologic data before replacing imaging with ctDNA can be considered. One of the most relevant settings in which ctDNA quantification may be of additional value is to differentiate between true progression and pseudoprogression in patients treated with immune checkpoint inhibitors [121]. Current studies are however limited by low patient numbers, Whether liquid biopsies can adequately result in refrainment from unnecessary treatment, costs, and potential side effects in patients with true progression on immunotherapy, while treatment is continued and eventually results in response in patients with radiologic pseudoprogression should be addressed in future studies.

### Liquid biopsies to evaluate mutations causing secondary resistance and tumor heterogeneity

Several studies describe the detection of new mutations during therapy implying progression on treatment and clonal heterogeneity of the tumors. In patients with NSCLC, it has been demonstrated that mutations which potentially cause therapy resistance can be detected in ctDNA during treatment with EGFR TKIs. For example, the well-known T790M mutation causing acquired resistance to EGFR inhibitors can be detected in ctDNA of lung cancer patients. Similarly, *PIK3CA* mutations causing endocrine therapy resistance in breast cancer patients can be detected in liquid biopsies [122].Thus, ctDNA could be a promising technique to identify patients at risk for disease progression and select or adjust systemic therapy accordingly to improve patient-tailored therapy. Aside from known resistance mechanisms, liquid biopsies may also aid to detect new mutations and give insight in other mechanisms of secondary resistance. Whether these detected mutations during the course of disease have a role in acquired therapy resistance and whether they could be targeted to overcome such treatment resistance must be assessed in larger clinical studies. In particular, assessment of the association between the golden standard (i.e., tumor biopsy) and detection of “new” mutations in plasma is essential.

### Other promising applications of liquid biopsies

Although beyond the scope of this review, there are various other areas of interest which may show clinical utility of liquid biopsies. Among these are (i) screening for early-stage cancer, (ii) to guide neoadjuvant therapy, (iii) as a surveillance tool after curative treatment, (iv) to assess recurrence risk after curative treatment and guide adjuvant therapy, and (v) liquid biopsies from other bodily fluids, such as urine or cerebrospinal fluid [104, 105].

## Conclusion

The aim of this review was to evaluate the clinical utility of ctDNA as marker for treatment response and follow-up in patients with mutation-driven solid malignancies during systemic therapy or after surgery. Although multiple studies show promising results for the utilization of ctDNA measurements in plasma to guide therapy decision-making and assess response in patients with solid tumors, larger prospective studies are needed. In order to be utilized as a blood-based marker, the association between ctDNA, tissue-based molecular analysis, tumor burden, radiologic response, and survival should be assessed for different tumor types, mutations, and targeted therapies individually.
